# The context for the thematic grouping of rare diseases to facilitate the establishment of European Reference Networks

**DOI:** 10.1186/s13023-016-0398-y

**Published:** 2016-02-24

**Authors:** Teresinha Evangelista, Victoria Hedley, Antonio Atalaia, Matt Johnson, Stephen Lynn, Yann Le Cam, Kate Bushby

**Affiliations:** John Walton Muscular Dystrophy Research Centre and MRC Centre for Neuromuscular Diseases, Institute of Genetic Medicine, Newcastle University, Newcastle upon Tyne, NE1 3BZ UK; European Organisation for Rare Diseases (EURORDIS), Paris, France

**Keywords:** Rare Diseases, European policies, European reference networks, Grouping of rare diseases

## Abstract

**Background:**

In the past few years there has been a political imperative driving the creation of European Reference Networks as these are considered a promising way to achieve equity in access to the most up to date medical care across Europe. The right to equity in the access to care was established by the directive of the European Parliament and of the Council on the application of patients' rights in cross-border healthcare. The particular situation for Rare Diseases whereby sharing of expertise can be regarded as especially valuable, as well as the work that is already in place in the networking of Rare Diseases experts means that Rare Diseases are considered excellent models for the development of European Reference Networks.

**Discussion:**

To be effective, a Rare Disease network should be based on the common effort of different stakeholders and be built on what is present in the community. European Reference Networks are an excellent model to overcome some of the specificities of rare diseases: scarcity of patients, resources and expertise. European Reference Networks with broad scope will allow the rare disease community the possibility of reaching a larger number of patients and more diversified rare diseases. The practical value of grouping rare diseases in broad networks is well demonstrated in different grouping systems present in Europe (EURORDIS grouping of diseases, “Les filières de santé maladies rares”, Orphanet classification and the UK Research Model).

**Summary:**

In this paper the authors, partners of EUCERD Joint Action, address some of the questions that surround the establishment of European Reference Networks. We will focus on how Rare Diseases could be efficiently grouped in order to constitute European Reference Networks and how they might be structured to allow each and every disease to benefit from networking.

## Background

Rare diseases (RD) are defined in the European Union as life-threatening or chronically debilitating conditions that affect less than 5 per 10 000 people. It has been well established in the academic and patient literature that the small number of patients and the geographic dispersion is an obstacle to the diagnosis, access to care, research and improvement of medical expertise. For a number of years it has been advocated that linking experts and pooling resources through networks would enable to overcome these obstacles, and significant expertise in networking across different RD groups has been established as demonstrated by the Orphanet Report: Series on Research Infrastructures for Rare Diseases in Europe (http://www.orpha.net/orphacom/cahiers/docs/GB/Research_Infrastructures_for_rare_diseases_in_Europe.pdf).

The European Union has tried to combat the lack of specific health policies for rare diseases in the different Member States, through the establishment of an overall strategy for Member States to support the equity in access to prevention, diagnosis and care for patients with a RD throughout the European Union.

In March 2011 the European Directive on the application of patient’s rights in cross-border healthcare [1] was adopted and established a legal framework for cross-border healthcare within the European Union (EU). The directive clarifies the rules of access to healthcare in a EU country different from the country of origin of the patient. It also clarifies the rules of reimbursement. One of the aims of the directive is to promote cooperation between the health systems of Member States including the establishment of European Reference Networks (ERNs). A network is by definition an association of individuals sharing common interests and providing mutual support and information. Due to its nature networks have the potential to cover large territorial areas. When we transpose these definitions to the organization of medical care, ERNs are an excellent model to overcome some of the specific problems of rare diseases: scarcity of patients, resources and expertise.

The recent publication of Implementing and Delegated Acts by the European Commission (EC) provides a framework for the creation of ERNs [2, 3]. Although RD are well placed to benefit from these instruments, it should be emphasised that ERNs were not specifically conceived for RD. It is also of note that the themes to be covered by ERNs are not prescribed in these acts.

In 2013 a study of the European Observatory on Health Systems and Policies [4] proposed some fundamental questions that should be tackled in the development of reference networks: which medical conditions or interventions should be addressed; what are the drivers and motivations for their creation; which regulatory, administrative and financial procedures are needed; and finally what are the impact and challenges for developing ERNs at European level.

The EUCERD Joint Action (EJA) is, since March 2012, assisting the EC with the formulation and implementation of its activities in the field of RD. Meantime, extensive work has been carried out by the EJA partners (John Walton Muscular Dystrophy Research Centre at Newcastle University and the European Organisation for Rare Diseases (EURORDIS)) with the European Union Committee of Experts in Rare Diseases/Commission Expert Group on Rare Diseases (EUCERD/CEGRD) on the generation of recommendations for RD ERNs. An important aspect with such a heterogeneous group of diseases as “rare diseases” is how to group them adequately to allow efficient and equitable functioning of RD ERNs.

In this paper we have focused on the process by which a decision was reached and adopted by the CEGRD as to how we could efficiently group RD in order to support the constitution of well-functioning ERNs.

## Discussion

### Healthcare networks

A significant body of work has been conducted around RD ERNs and the likely benefits have been well documented [1, 5–7]. ERNs for RD can be established considering different aspects, such as: disease frequency and prevalence, cost-effectiveness, or the need to reach sufficient numbers of patients to increase expertise and deliver the best quality of care. However, independently of the established priority, a RD ERN to be effective should be based on the common effort of different stakeholders and build on what is already present in the community. The principles for the establishment of RD ERNs were enshrined in the EUCERD recommendations on this topic in January 2013 [8].

The concept behind the implementation of ERNs as a model for healthcare provision has clearly evolved from the pure aim to improve cost-effectiveness to encompass improving quality of care and equity in access to healthcare. This shift makes it relevant to consider that the priority around RD ERNs should be reaching sufficient numbers of patients to increase expertise and deliver the best quality of care. Therefore, one of the main challenges for the society in general and for the rare diseases stakeholders in particular is to determine how ERNs should be organized i.e. how many should be created and how should different diseases be grouped in order to achieve the above-mentioned priority.

### Why organize ERNs around groups of diseases?

A network, in social sciences, is defined as an association of individuals sharing common interests and providing mutual support and information [9]. Networks have the potential to achieve a large coverage depending on the number of members involved. When we transpose these definitions to the organization of medical care, ERNs are an excellent model to overcome some of the specific problems of rare diseases: scarcity of patients, resources and expertise. ERNs with a broad scope will allow the rare disease community the possibility of reaching a larger number of patients and a more diverse range of rare diseases.

With respect to existing networks, ERN status will need to represent a clear added-value such as: easier adoption and spread of innovations in medical science and health technologies; facilitation of medical training; faster dissemination of agreed standards of care and general knowledge in the area of expertise; and increased profile and recognition of the participants. ERNs will attract the best experts and more patients and although they are supposed to facilitate cross-border health care it is expected that the pooling of resources and the e-Health solutions will decrease the burden of travelling for the patients. Another expected though not explicit benefit is that the Networks and their members will be in a better position to apply for research funding and further develop and accelerate basic and translational research.

Existing networks in the RD field have arisen “ad hoc” in response to specific funding calls either from DG Sanco (now Santé) or from DG research. The diseases covered by these networks are often of low/very low prevalence and the networks highly focussed. The aims of the networks are highly variable and their sustainability has been a major issue (Table 1). No mechanism has yet been in place to ensure that all or even a majority of RD patients might have access to a network for their particular disease. In the process of drafting and adopting the EUCERD recommendations on RD ERNs, this issue of inclusivity was debated in some detail. Patients’ organisations such as EURORDIS strongly advocated that ERNs should be inclusive and should not be created for every single RD, on the grounds that this would leave many patients without an ‘umbrella’ ERN [10]. Creating 6–8000 individual ERNs (as many as the estimated number of rare diseases) would be impossible, and not desirable from a clinical perspective.

**Table 1:** Existing networks/projects in the rare disease field supported by EU funding

Group of diseases	Networks/Projects
Rare cardiac diseases	CHD - Congenital Heart Defects
Rare connective tissue and musculoskeletal diseases	PRINTO - paediatric rheumatology international trials organisation ESDN: European Skeletal Dysplasia Network
Rare hereditary metabolic disorders	E-IMD - European registry and network for Intoxication type Metabolic Diseases EUROGLYCANET CDG: Congenital Disorders of Glycosylation
Rare haematological diseases	EPNET – European Porphyria Network EN-RBD - Rare Bleeding Disorders Paediatric Hodgkin's lymphoma Network ENERCA - European Network for Rare and Congenital Anaemias EUHANET - Haemophilia and the rare congenital deficiencies of other coagulation factors
Rare immunological and auto- inflammatory diseases	EURO-HISTIO-NET - A reference network for Langerhans cell histiocytosis and associated syndrome
Rare cancers	ExPO-r-NeT - European Expert Paediatric Oncology Reference Network for Diagnostics and Treatment RARECARENet - Information network on rare cancers
Rare hepatic diseases	EUROWILSON: European network on Wilson disease
Rare neurological diseases	NEUROPED - European Network of Reference for Rare Paediatric Neurological Diseases LEUKOTREAT: Leukodystrophies EUROSCA: European integrated project on spinocerebellar ataxias E-Pilepsy - Refractory Epilepsy
Rare Neuromuscular diseases	Care-NMD - Improving care for Duchenne muscular dystrophy TREAT-NMD – Neuromuscular network
Rare skin disorders	TAG - Together Against Genodermatoses GENESKIN: European network on rare genetic skin diseases
Rare Pulmonary diseases	ECORN CF – Expert Advice on Cystic Fibrosis PAAIR - Patient Associations and Alpha1 International Registry ENCE CF-LAM-LTX - European networks of centres of expertise for CF (Cystic Fibrosis), LAM (Lymphangioleiomyomatosis), and LTX (Lung Transplantation) EUROCARE CF - Cystic Fibrosis
Rare malformations and developmental anomalies	DYSCERNE - Rare Dysmorphic Syndromes EUROCRAN - Craniofacial anomalies
Rare Kidney diseases	EuroCYST initiative - Polycystic Kidney Diseases

Following the publication of the delegating and implementing acts, further discussion within the CEGRD indicated that more guidance was needed on the grouping of diseases into ‘families’ that can be addressed by comprehensive ERNs as a realistic and constructive approach.

### Grouping exercise

In order to be successful, ERNs need to take into consideration existing national practices and networking systems which entails both opportunities and challenges. Existing networks and national healthcare authorities need to be made aware of the economical, scientific and patient care benefits of joint versus single disease networks.

The EUCERD Recommendations on Rare Diseases European Reference Networks (RD ERNS), published on 31 January 2013, aimed at provide guidance to the Member States and the EC on the criteria needed to be fulfilled to establish RD ERNs [8]. These recommendations were generated taking on board the results achieved by pilot ERNs funded through DG Sanco or DG Research between 2008 and 2013.

After the publication of the Delegated and Implementing Acts in March 2014 the EJA conducted a mapping exercise to explore the areas where the previous recommendations needed further attention from the EC Expert Group on Rare Diseases. That mapping exercise was the basis for an addendum to the EUCERD recommendations. Based on the content of the EUCERD Recommendations and the content of the Acts the Addendum to the Recommendations suggested an illustrative grouping of RD as a rational approach to RD ERN planning and to ensure coverage of all RD.

We based our exercise about the grouping of rare diseases on several possible models such as: the alignment of diseases by clinical area (e.g. Neurology, Neuromuscular, Psychiatry, Skin, Kidney, etc.), by clinical group (e.g. Genetic Disorders, Metabolic Disorders, Epilepsy, Oncology), clinical intervention area (e.g. Transplantation, Gene Therapy, Radiotherapy), shared molecular aetiology [11] (e.g. Underlying disease mechanism or pathway such as fibrosis or inflammation) or mixed models.

Several successful examples of centres of expertise and international networks for RD already exist, which may be viewed as concrete, trusted ‘solutions’ in their field of expertise and should be approached as case-studies from which one can derive important lessons for future ERNs. These tend to be grouped by clinical area or clinical grouping (e.g. rare anaemias, neuromuscular diseases, metabolic diseases, rare epilepsies, etc.).

An ERN based on a specific treatment area, such as for example Gene Therapy, although attractive when considering clinical trials or therapy development would inevitably leave numerous rare disease patients without a “home”. This model would obviously be involved with different clinical areas and could be regarded as transversal, cutting across numerous medical specialties. Such a transversal ERN, could not replace ERNs based on clinical areas or clinical groups and would have to co-exist alongside these.

ERNs based on shared molecular mechanisms would, as the former model, be a way of addressing problems related with clinical trials and drug development. Drugs that target a molecular pathway that is common to multiple diseases can, in principle, be used to treat more than one disease. However, though the concept is interesting in particularly from a research perspective, such an ERN might prove rather restrictive and too “single issue”, especially given the multidisciplinarity inherent in the expectations asked of an ERN.

We therefore compared five examples of disease grouping based on clinical areas/groups (the Classical Medical Ontology; the Orphanet classification; the EURORDIS preliminary proposal for grouping of rare diseases; the French filières; and the UK Research Model as established by the NIHR Translational Research Collaboration on RD). This comparison enabled us to propose a preliminary strategy for grouping rare diseases based on the merging of the common items. The classical medical ontology is the one used in Internal Medicine textbooks and is usually organised according to major organs and systems. Although incomplete for RD, it is widely used for teaching and is imbedded in the physicians’ way of thinking. A well-known example is the classification used in Harrison's Principles of Internal Medicine [12].

The Orphanet classification of rare diseases is structured around 116 groups of diseases. For the purposes of creating ERNs, 116 ‘categories’ would be excessive; however, in terms of thematic grouping Orphanet proposes 31 RD categories [http://www.orphadata.org/cgi-bin/inc/product3.inc.php]. It is a useful scientific classification; however, when dealing with ERNs there are some overlaps and some redundant groups. If, for instance, we consider the broad group of “Rare genetic diseases” should sickle cell anaemia be in the scope and expertise of an ERN for rare genetic diseases or for rare haematological diseases? Is it practical to have an ERN devoted to rare intoxications defined by Orphanet as rare intoxications due to medical products, or should these be incorporated in ERNs dealing with, for instance cardiac disorders for digitalis intoxication or with oncology for cytostatic intoxication?

The EURORDIS proposal, presented at the workshop “Rare Disease European Reference Networks (RD ERNs) and the use of structural funds to support activities for RD” held in Rome on the 28-29th October 2014 organises ERNs by clinical area and was based upon research undertaken by EURORDIS collaborators. This was an outline document, kindly made accessible to us by EURORDIS, which drafted groupings to facilitate discussion with the Council of National Alliances, Council of European Federations and EURORDIS members (Table 2). The Second French National Plan for Rare Diseases mandated the creation of ‘filières de santé maladies rares’. These “French national rare disease healthcare networks” aim to coordinate all the missions and activities of groups of centres of expertise and related competence centres in charge of coherent groups of related rare diseases. At present 23 official networks were created [http://www.sante.gouv.fr/les-filieres-de-sante-maladies-rares.html]. The ‘Filières de santé maladies rares’ were adequately based on what was already in place in the French health system. The main problem stays when we try to extrapolate these country-specific pathways to a broader coverage. Also, the ‘themes’ range from relatively broad (e.g. inborn metabolic diseases or developmental anomalies and malformations) to disease-specific (e.g. Amyotrophic Lateral Sclerosis). Given the lack of therapeutic options for the vast majority of the 6–8000 rare diseases, there is a necessary link between research and clinical care provision; therefore, we have also considered in our analysis the example of how RD have been grouped by the UK National Institute for Health Research (NIHR) Translational Research Collaboration in Rare Diseases (Table 3).

**Table 2:** EURORDIS Proposal for grouping of diseases for ERNs

1. Undiagnosed Conditions RD ERN	
2. Immunologically-mediated and Systemic RD ERN	
3. Cardio-Vascular Diseases RD ERN	
4. Malformations/Medical Genetics/Neuropaediatrics RD ERN	
5. Dermatological diseases RD ERN	
6. Endocrinal Diseases RD ERN	
7. Hepatic gastroenterological and Severe Intestinal Disorders RD ERN	
8. Non-Malignant Haematological Diseases RD ERN	
9. Hereditary Metabolic Diseases RD ERN	
10. Neurological Diseases RD ERN	
11. Neuromuscular RD ERN	
12. Pulmonary RD ERN	
13. Kidney RD ERN	
14. Connective Tissue Framework and Specialist Rheumatology Diseases RD ERN	
15. Head & Neck Malformations RD ERN and Sensory Diseases RD ERN (including rare ophthalmological, congenital and genetic disease)	
16. Cancers RD ERN	
17. Other Rare Diseases RD ERN	
18. Rare Orthopaedic diseases including Complex Spinal Disorders RD ERN	
19. Women, neonatal and children RD ERN

**Table 3:** UK grouping of rare diseases for research purposes

1. Cancer	
2. Cardiovascular	
3. Dementia and Neurodegenerative	
4. Eye Disease	
5. Gastrointestinal	
6. Immunological Disorders	
7. Metabolism	
8. Musculoskeletal Disorders	
9. Neuromuscular Disorders	
10. Non-Malignant haematology	
11. Paediatric (cross-cutting)	
12. Renal Disease	
13. Respiratory Disease	
14. Skin	

Legend: example of how RD have been grouped by the UK National Institute for Health Research (NIHR) - Translational Research Collaboration in Rare Diseases

**Table 4:** ORPHANET Classifications

Rare cardiac diseases	
Developmental anomalies during embryogenesis	
Inborn errors of metabolism	
Rare gastroenterological diseases	
Rare neurological diseases	
Rare abdominal surgical diseases	
Rare hepatic diseases	
Rare respiratory diseases	
Rare urogenital diseases	
Rare surgical thoracic diseases	
Rare skin diseases	
Rare renal diseases	
Rare eye diseases	
Rare endocrine diseases	
Rare haematological diseases	
Rare immunological diseases	
Rare systemic and rheumatologic diseases	
Rare odontological diseases	
Rare circulatory system diseases	
Rare bone diseases	
Rare otorhinolaryngological diseases	
Rare infertility disorders	
Rare tumours	
Rare infectious diseases	
Rare intoxications	
Rare gynaecological and obstetric diseases	
Rare surgical maxillo-facial diseases	
Rare allergic disease	
Teratological disorders	
Rare cardiac malformations	
Rare genetic diseases	

Legend: Each of these headings represents a separate and more exhaustive classification. (http://www.orphadata.org/cgi-bin/inc/product3.inc.php)

### Conclusion

After analysing these different models we have tried to merge the common lines having in consideration that the grouping strategy has to incorporate the need of having healthcare providers with different roles/expertise in any network, the fact that a healthcare provider may take part in different ERNs, the need to have a significant collaboration across ERNs as well as within, and that fluidity and inter-communication between ERNs should be ingrained in the structure of the network. Another important question that was debated was whether a dedicated ERN for undiagnosed patients would be feasible or desirable. Here the distinction between truly undiagnosed patients (those in whom a precise diagnosis is not possible and who may require access to research programmes for a diagnosis to be achieved, such as is available via the NIH undiagnosed scheme in the USA) and the patients who are known to have a particular type of disease but not yet a precise diagnosis, needs to be made. As an alternative to a dedicated ‘undiagnosed’ RD ERN, it is suggested that each ERN should maintain a ‘forum’ for undiagnosed patients suspected to fall within their area of expertise. Considering what was stated about how RD ERNs should be structured, the necessity to encompass all rare disease patients and the merging of the different models we have studied, we suggest the constitution of 22 broad groups (Table 5). This list was posteriorly presented to the Commission Expert group on Rare Diseases for discussion. Some changes were suggested and the final grouping (Table 6) was published as part of the Addendum to the EUCERD recommendations of January 2013 [8].

**Table 5:** Grouping of RD for Future ERNs, based on areas of overlap in the systems outlined in the text

1. Rare cardiac diseases (with rare cardiac malformations ERN included or separate)	
2. Rare connective tissue and musculoskeletal diseases	
3. Rare hereditary metabolic disorders	
4. Rare haematological diseases	
5. Rare diseases of brain development and rare intellectual disabilities	
6. Rare auto-immune and auto inflammatory diseases	
7. Rare cancers	
8. Rare hepatic diseases	
9. Rare gastrointestinal diseases	
10. Rare neurological diseases	
11. Rare neuromuscular diseases	
12. Rare skin disorders	
13. Rare pulmonary diseases	
14. Rare malformations and developmental anomalies	
15. Rare endocrine diseases	
16. Rare urogenital diseases	
17. Rare renal diseases	
18. Rare multi-systemic vascular diseases	
19. Rare head and neck diseases	
20. Rare gynaecological and obstetric diseases	
21. Rare eye diseases	
22. Rare bone diseases	

Legend: The present list was proposed by the EUCERD Joint Action team and was subsequently submitted to the Commission Expert group on Rare Diseases for discussion

**Table 6:** Grouping rare diseases in thematic networks

Rare immunological and auto-inflammatory diseases	
Rare bone diseases	
Rare cancers and tumours	
Rare cardiac diseases	
Rare connective tissue and musculoskeletal diseases	
Rare malformations and developmental anomalies and rare intellectual disabilities	
Rare endocrine diseases	
Rare eye diseases	
Rare gastrointestinal diseases	
Rare gynaecological and obstetric diseases	
Rare haematological diseases	
Rare craniofacial anomalies and ENT (ear, nose and throat) disorders	
Rare hepatic diseases	
Rare hereditary metabolic disorders	
Rare multi-systemic vascular diseases	
Rare neurological diseases	
Rare neuromuscular diseases	
Rare pulmonary diseases	
Rare renal diseases	
Rare skin disorders	
Rare urogenital diseases	

Legend: Thematic grouping of networks published in RARE DISEASE EUROPEAN REFERENCE NETWORKS: ADDENDUM TO EUCERD RECOMMENDATIONS OF JANUARY 2013

**Fig. 1:**
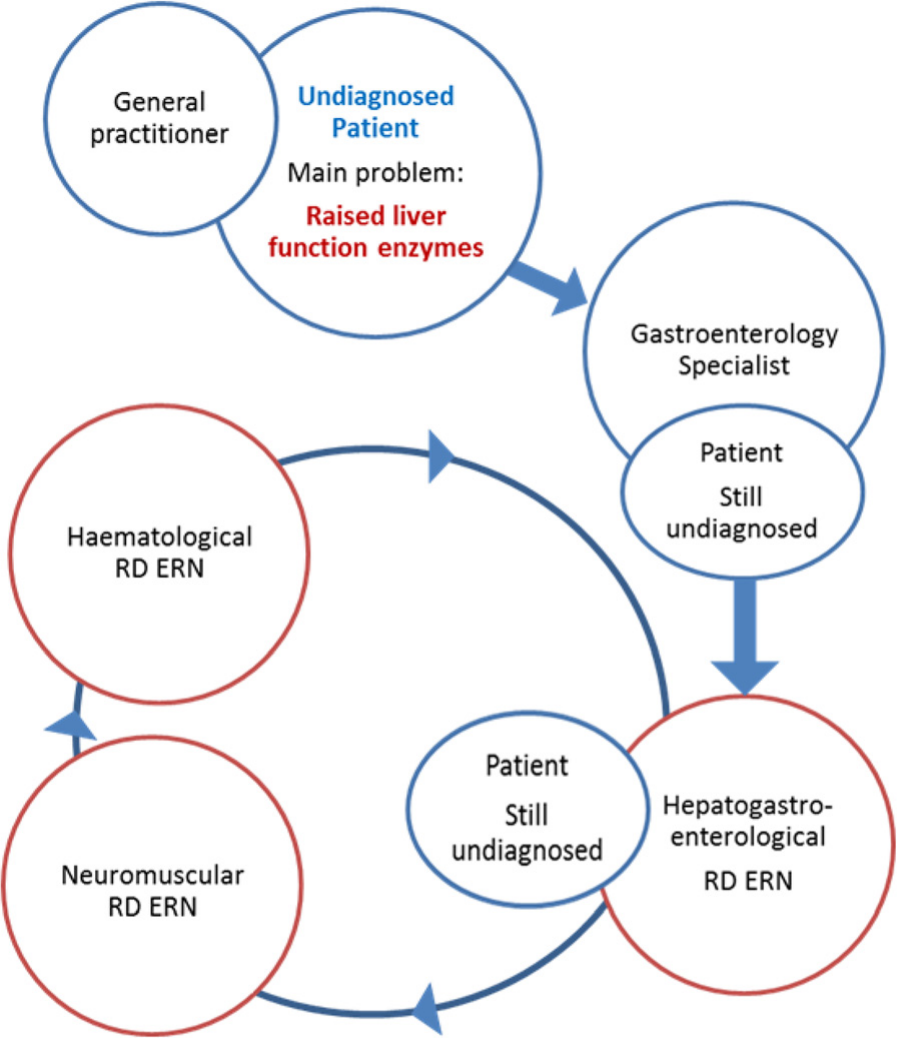
Possible pathway for undiagnosed patients in the presence of flexible networks

As with the previously described models, the proposed model is not perfect. However some of the difficulties can be overcome as long as the ERNs are organized as flexible structures with capacity to adjust to the reality and maintain enough plasticity to share patients amongst them, in particular the undiagnosed ones (Fig. 1).

## Summary

ERNs must be sufficiently broad and flexible in their scope to adjust to real life. When establishing ERNs one should aim to encompass all rare disease patients including those without a precise diagnosis. To achieve these goals ERNs should not be considered as static, nonflexible structures. They should take under their umbrella those patients that, according to the state of the art, are considered undiagnosed but whose clinical manifestations fall in the respective area of expertise. ERNs should aim to interact with other ERNs, should share common ontologies and coding systems, and should have inter-operational IT technologies. Despite variation in the details of some of the models, a group of approximately 22–25 clinical disease areas form the core of the different systems and could be a starting point for a cohesive European approach.

As with social networks [9] a RD ERN should have a cooperative structure with decentralized authority. It has to build on critical mass and to develop collective intelligence. Collective intelligence principles, a collective rather than an hierarchical approach to decision making and development of intellectual content, are not strange to the healthcare systems and have been seen as a way of maintaining access to services that otherwise due to the scarcity of resources the healthcare system would not be able to offer [13]. For RDs, ensuring the integration of these core features poses unique challenges, yet, simultaneously promises to generate unique added-value. For example, in terms of ‘collective intelligence’, RD ERNs will support expert collaboration and communication. It is also known that in the past a lack of ‘critical mass’ has hindered RD research and healthcare provision, ERNs will facilitate this by bringing together patients and healthcare providers in a sustainable structure with a clear position within healthcare policy.

## Abbreviations

RD: Rare Diseases
EU: European Union
ERNs: European Reference Networks
EC: European Commission
EUCERD: European Union Committee of Experts in Rare Diseases
CEGRD: Commission Expert Group on Rare Diseases
EJA: EUCERD Joint Action
EURORDIS: European Organisation for Rare Diseases
